# The SDH mutation database: an online resource for succinate dehydrogenase sequence variants involved in pheochromocytoma, paraganglioma and mitochondrial complex II deficiency

**DOI:** 10.1186/1471-2350-6-39

**Published:** 2005-11-16

**Authors:** Jean-Pierre Bayley, Peter Devilee, Peter EM Taschner

**Affiliations:** 1 Department of Human Genetics, Center for Human and Clinical Genetics, Leiden University Medical Center, P.O. Box 9503, 2300 RA, Leiden, The Netherlands; 2 Department of Pathology, Leiden University Medical Center, P.O. Box 9600, 2300 RA, Leiden, The Netherlands

## Abstract

**Background:**

The SDHA, SDHB, SDHC and SDHD genes encode the subunits of succinate dehydrogenase (succinate: ubiquinone oxidoreductase), a component of both the Krebs cycle and the mitochondrial respiratory chain. SDHA, a flavoprotein and SDHB, an iron-sulfur protein together constitute the catalytic domain, while SDHC and SDHD encode membrane anchors that allow the complex to participate in the respiratory chain as complex II. Germline mutations of SDHD and SDHB are a major cause of the hereditary forms of the tumors paraganglioma and pheochromocytoma. The largest subunit, SDHA, is mutated in patients with Leigh syndrome and late-onset optic atrophy, but has not as yet been identified as a factor in hereditary cancer.

**Description:**

The SDH mutation database is based on the recently described Leiden Open (source) Variation Database (LOVD) system. The variants currently described in the database were extracted from the published literature and in some cases annotated to conform to current mutation nomenclature. Researchers can also directly submit new sequence variants online. Since the identification of SDHD, SDHC, and SDHB as classic tumor suppressor genes in 2000 and 2001, studies from research groups around the world have identified a total of 120 variants. Here we introduce all reported paraganglioma and pheochromocytoma related sequence variations in these genes, in addition to all reported mutations of SDHA. The database is now accessible online.

**Conclusion:**

The SDH mutation database offers a valuable tool and resource for clinicians involved in the treatment of patients with paraganglioma-pheochromocytoma, clinical geneticists needing an overview of current knowledge, and geneticists and other researchers needing a solid foundation for further exploration of both these tumor syndromes and SDHA-related phenotypes.

## Background

Pheochromocytoma is a tumor of the sympathetic nervous system, arising in the chromaffin cells of the adrenal medulla. Tumors also occur in related sympathetic ganglia and are properly described as paragangliomas, by anatomical location. Both these tumor types are generally catecholamine secreting. Paragangliomas of the head and neck (HN PGL) are related tumors which are rarely catecholamine secreting, arise in the parasympathetic ganglia, most commonly in the carotid body, but also frequently found as vagal and jugulotympanic tumors. The hereditary element in these tumors has long been recognized [1] and a putative genetic locus for PGL1 was first mapped in 1992 by Heutink et al. [2,3]. The identification of PGL1 followed, when Baysal et al. [4] reported germline mutations in the gene encoding succinate dehydrogenase, subunit D (SDHD) in PGL1-linked families. A candidate gene approach quickly identified germline mutations in the other SDH subunits, SDHC (PGL3) [5] and SDHB (PGL4) [6]. It is now recognised that SDHD and SDHB, together with the VHL, RET and NF1 genes, play a major role in the hereditary forms of both pheochromocytoma and paraganglioma [7]. In contrast, mutations of SDHA result in a range of clinical phenotypes, including Leigh syndrome, but have never been reported in relation to HN PGL or pheochromocytoma.

The SDH genes encode subunits of the heterotetrameric succinate dehydrogenase complex, a component of both the mitochondrial-respiratory chain (complex II) and the Krebs cycle. SDHA (Ch5p15) and SDHB (Ch1p36) encode the two catalytic subunits, the flavoprotein and the iron-sulfur protein respectively; SDHC (Ch1q21) and SDHD (Ch11q23) encode transmembrane proteins that anchor complex II in the inner mitochondrial membrane, and contain a ubiquinone binding site. The SDHA gene consists of 15 exons, with a second isoform [8] and at least one pseudogene (Ch3q29) present in the genome. SDHB has eight exons and no known pseudogenes, while SDHC covers six exons and has three candidate pseudogenes. SDHD has four exons and six reported intronless pseudogenes [9].

## Construction and content

The SDH mutation database [10] is based on the recently described Leiden Open (source) Variation Database (LOVD) system [11]. Researchers may submit new sequence variants online and submitters can access and edit their personal data at any time. During the submission procedure researchers will be asked to fill in several fields on the submission form, providing those data that are deemed absolutely essential for mutation databases by the Human Genome Organization Mutation Database Initiative. These include a patient ID, an exact molecular description of the variant (DNA-level), and details about the source of the material and detection method used. Mutations are described in accordance with the recommendations of the Human Genome Variation Society (HGVS), update August 2004, and it should be noted that the current nomenclature can differ significantly from previous versions [12] and from that used in the literature.

Newly submitted data are forwarded automatically to the database curators and each variant is given a unique identifier as recommended by Claustres et al. [13]. After the curator's approval the new variant is automatically included in the database and all connected web pages are updated instantly.

The SDH database [10] includes (as of September 2005) 120 variants of which 98 are thought to be pathogenic and 22 non-functional variants (polymorphisms). The most common types of mutations are missense and nonsense, with relatively frequent small deletions and small insertions. Missense mutations are the most common form but still occur at half the expected relative frequency when compared to the mutation summary of the Human Gene Mutation Database. Reports of large deletions are sparse but this may simply reflect limited effort in this direction to date.

Inclusion of sequence variants in the SDH mutation database does not imply that there is convincing evidence for pathogenicity. Please refer to the disclaimer on the website.

Within the SDH database, all variants that disrupt the reading frame, affect highly conserved residues or disrupt the consensus donor or acceptor splice sites (GT-AG), and are not found in healthy controls, can be considered to be pathological.

In the case of non-conserved missense variants and potential splice site mutations that do not disrupt the consensus splice sites, a designation of "unclassified variants" (UV) should eventually be developed. The CMGS guidelines (CMGS Best Practice Guidelines – Molecular Genetics Service Testing for HNPCC) suggest that several lines of supporting evidence can be valuable, including screening a panel of normal DNA from 50–200 individuals to rule out a common polymorphism, describing the nature of the amino acid change (conservative or non-conservative) and the significance of the position in the coding sequence (evolutionarily conserved or known functional domain). Confidence increases if the mutation has been previously described, in several families, and if it segregates with the disease within the family. However none of these factors can be taken as definitive and each variant must be considered on its merits. Unfortunately, most mutations are currently reported without this accompanying analysis, and many have been identified in a single case or family. Thus caution should be exercised when attempting to derive clinically relevant information from the database, and all the evidence in the database and any additional data must be carefully weighed by users.

Polymorphisms, including intron variants, synonymous (silent) variants, nonsynonymous missense variants found in a healthy control panel (e.g. p.His50Arg of SDHD) and potential non-consensus splice site mutations but without evidence for transcript rearrangements are included in the database as such unless accompanied by clear evidence of pathogenic potential.

The database includes a brief description of the tumor types presenting in carriers, and an explanation of the abbreviations is included. Under "remarks" the country of origin of patients (if reported), or of the study itself, is included to aid the identification of founder mutations, already known to play a major role in the incidence of hereditary HN PGL in the Netherlands. In addition other relevant information, such as the number of healthy controls tested for the variant, and further supporting evidence, is described.

Other columns detail the number of reported familial 'carriers or cases' (not generally distinguishable in current literature) and the number of 'sporadic' cases (those with no known familial antecedents). For further details, the 'full legend' feature can be consulted.

While the description of many mutations reported in the literature remains unchanged, many others have been adapted to the standardized nomenclature, or in the case of frameshifts, have been fully annotated in the long version which includes the length of the additional amino acid chain that results. That a standardized nomenclature provides clarity is seen in several cases where authors have reported "novel" mutations, when in fact the same mutation had already been described but using a different sequence annotation or nomenclature. To avoid confusion and aid reference to original sources, the annotation used in the original report is included in the 'original description' column.

## Utility

The SDH mutation database is organized in a gene and exon centered fashion, and as such will be particularly useful to clinical geneticists, providing an up-to-date overview of all known SDH mutations. The database will also be of interest and useful to general and specialist physicians involved in the care and treatment of patients with pheochromocytoma, paraganglioma and complex II deficiencies including Leigh syndrome.

**Figure 1:**
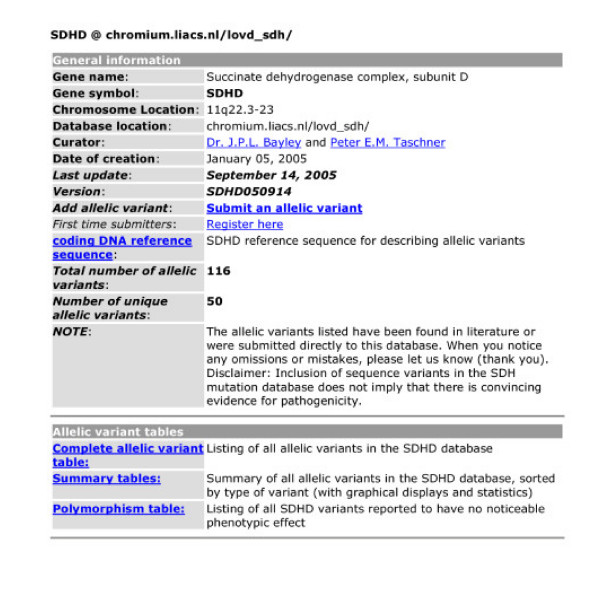
The introductory page of the SDHD mutation database. In addition to summary tables, various search options are also available.

**Figure 2:**
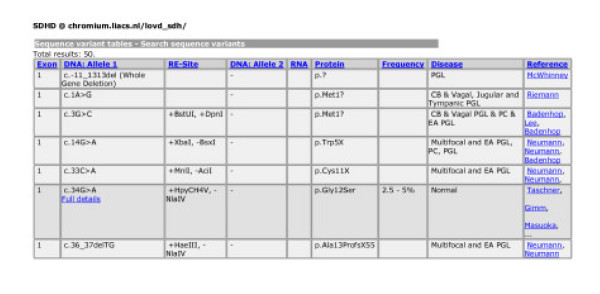
A partial overview of the SDHD table of allelic variants.

The four SDH genes each have a separate database with a summary page listing general gene and database information and providing access to the tables containing the allelic variant information and several search options (Fig. 1). The complete allelic variant table contains the sequence variations ordered by position relative to the cDNA reference sequence (Fig. 2).

Since the first description of mutations of SDHD, SDHB and SDHC in paraganglioma and pheochromocytoma, a series of reports have appeared describing a total of 47 distinct mutations in SDHB and 42 in SDHD (Table 1). While patterns already seem to be emerging (discussed below), given the number of SDHB and SDHD mutations currently known, it is important to realize that any conclusions must be seen as provisional, and only an expansion of the database will allow more definite conclusions to be drawn.

### Phenotype

Patients with mutations of SDHB and SDHD have recently been shown to display distinct clinical phenotypes [14]. This finding is clearly reflected in the SDH database (Fig. 2).

### Missense vs. nonsense

An interesting feature of the current SDHB-SDHD mutation spectrum is the difference in the frequency of missense mutations in contrast to truncating mutations (nonsense, frameshift, splice site and major deletions). While missense mutations are relatively more common in SDHB, truncating mutations are more frequent in SDHD (Table 2). The larger number of missense mutations observed in SDHB suggests that the SDHB protein is under greater structural constraint than the SDHD protein, which is also reflected by a higher degree of conservation for SDHB. Thus the weaker conservation of SDHD may allow more non-deleterious missense changes and require frameshift and truncating mutations before a pathogenic effect is seen. The missense mutations of SDHB cluster in several regions, including the iron-sulfur clusters, while the missense mutations of SDHD seem to cluster around the three transmembrane domains.

### Exon distribution

**Figure 3:**
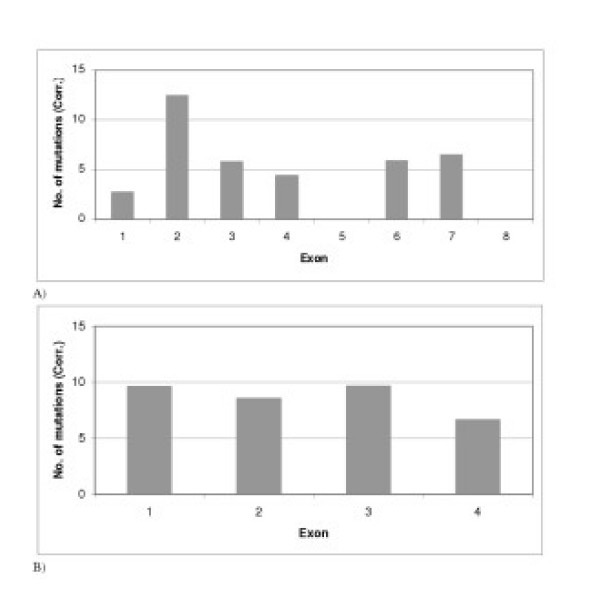
The distribution of mutations affecting the coding region (corrected for length of the exon) for SDHB (n = 43) is significantly different from an expected even distribution (ChiSq, p = 0.002) and B) for SDHD (n = 40), mutations occur approximately equally in all four exons (p = 0.99).

A further striking difference is in the distribution of mutations over the exons of the respective genes. SDHD mutations are found evenly distributed over the four exons while mutations of SDHB are concentrated in certain exons, most notably exon 2 (16 mutations) and are entirely absent from exons 5 and 8 (Fig. 3).

### SDHA and SDHC

A striking discrepancy has arisen between the numbers of mutations reported in the SDHB and SDHD genes and those of SDHA and SDHC. The divergent phenotype associated with SDHA, Leigh syndrome or Leigh-like symptoms, and the predominantly recessive inheritance pattern may partly explain the paucity of reported mutations in this gene. More curious, however is the small number of SDHC mutations reported, despite inclusion of SDHC in many of the screening efforts of paraganglioma/pheochromocytoma patients [15-22]. To date 42 different pathogenic mutations have been reported to affect the 159 amino acid SDHD protein while only four have been found affecting the 169 amino acids of the SDHC protein.

## Discussion

The SDH database provides the only complete and up-to-date overview of all disease-related gene variants reported in SDH subunits. In addition, columns describing supporting evidence and clinical features provide a starting point for further exploration of the possible relevance of each variant. An example of utility is the clear divergence in clinical phenotypes between SDHB and SDHD (Fig. 2), supporting the conclusions of Neumann *et al *[14]. In the future, we hope to link this gene-centered database to clinically-oriented databases of HN PGL and SDH-related pheochromocytoma, allowing closer gene-phenotype and mutation-phenotype correlations.

A striking feature of the SDH database is the eight-fold greater number of reported mutations in SDHB and SDHD compared to SDHA and SDHC. This is perhaps unexpected in that all are subunits of a single protein complex, and, in yeast, mutation in any one of the four genes leads to loss of SDH function and an inability to grow by respiration [23]. An explanation, in the case of SDHA, may be the presence in the genome of a second isoform [8] and the fact that known human SDHA mutations do not lead to complete loss of the electron transfer function [24]. The discrepancy in mutation frequency in SDHC and SDHD is more difficult to explain due to our present view of the structural and functional equivalence of these proteins. At least eight studies have failed to find mutations of SDHC in paraganglioma and pheochromocytoma patients. Although the SDHC gene, on chromosome 1, might be more commonly affected by major deletions, it is possible that another genetic mechanism might explain the discrepancy. Recent evidence suggests that additional genetic factors located on chromosome 11 may, together with SDHD, play a role in the tumorigenesis of HN PGL and pheochromocytoma [25,26].

## Conclusion

The variation in both phenotypes and mutation frequencies amongst the four subunits of complex II and our current inability to provide an explanation illustrates how little we still know about both the diseases in question and the biological functions of complex II. Knowledge brings understanding and a database of all known mutations in the genes encoding SDHA, B, C, and D will, we believe, represent a valuable tool and resource for both clinicians involved in the treatment of paraganglioma and pheochromocytoma patients, clinical geneticists needing a overview of current knowledge, and geneticists and other researchers needing a solid foundation for further exploration of the genetic aspects of these tumor syndromes, SDH function, and SDHA related phenotypes.

## Availability and requirements

The SDH mutation database is freely accessible to all at .

All researchers may submit new sequence variants online (after registration – to collect contact information for reference purposes and clarification of submitted details, as well as to assign a login name and password).

## Competing interests

The author(s) declare that they have no competing interests.

## Authors' contributions

JPB collected, edited and analysed the data, wrote the manuscript, and is the principal database curator. PT collected, edited and analysed the data, designed and implemented the database, co-wrote the manuscript, and is the database manager. PD contributed to the conception of the database, the collection and analysis of the data, and read and revised the manuscript. All authors read and approved the final manuscript.

## Pre-publication history

The pre-publication history for this paper can be accessed here:



## Figures and Tables

**Figure 4:**
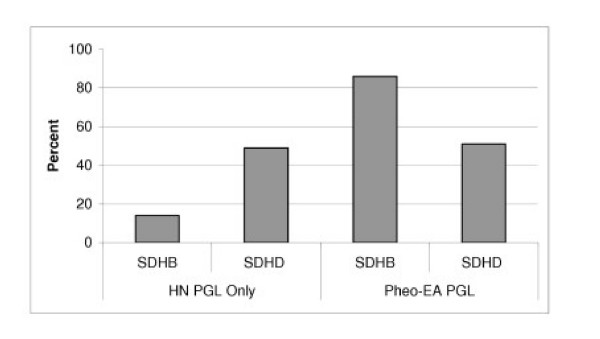
The relative occurrence of SDHB vs. SDHD mutations in patients with HN PGL only or pheochromocytoma/extra-adrenal paraganglioma with or without HN PGL.

**Table 1:** A summary of all allelic variants in the SDH mutation database as of September 2005.

	Gene				
Sequence Variant	SDHA	SDHB	SDHC	SDHD	Total
Total – Sequence Variants	5	60	5	50	120
Total – Mutations	5	47	4	42	98
Missense	4	19	1	11	36
Nonsense (incl. start codon)	1	8	1	11	21
Small Insertions <10 bp	0	4	0	3	7
Small Deletions <10 bp	0	9	0	13	22
Splice site	0	6	1	2	9
Large Insertions/deletions	0	1	1	2	4
Total – Polymorphisms	0	13	1	8	22
Synonymous (Silent)	0	3	0	4	7
Non-Synonymous	0	1	0	2	3
Promoter or Intron polymorphisms	0	9	1	2	12

**Table 2:** Differing frequencies of missense mutations vs. truncating mutations (nonsense, frameshift, splice site and major deletions) in SDHB and SDHD.

Gene	SDHB	%	SDHD	%
Type of Variant				
Missense	19	40	11	26
Truncating (incl. splice site)	28	60	31	74
Total	47		42	
